# Reverse engineering in medical application: literature review, proof of concept and future perspectives

**DOI:** 10.1038/s41598-024-74176-z

**Published:** 2024-10-09

**Authors:** Yosef Wakjira, Navaneethan S. Kurukkal, Hirpa G. Lemu

**Affiliations:** Faculty of Science and Technology, Department of Mechanical and Structural Engineering and Materials Science, University of Stavanger, Kjell Arholms Gate 41, 4021 Stavanger, Norway

**Keywords:** Reverse engineering, 3D scanning, Imaging techniques, Healthcare, Reconstruction, Engineering, Biomedical engineering, Mechanical engineering

## Abstract

Reverse engineering, a process of extracting information or knowledge from existing objects or systems, has gained significant attention in various fields, including medicine. This article presents a comprehensive literature review and a proof of concept on the application of reverse engineering in the medical field. The review particularly focuses on the reverse engineering process, available technologies, and their specific relevance to the medical domain. Various imaging techniques, such as computed tomography and magnetic resonance imaging, are discussed in respect of their integration with reverse engineering methodologies. Furthermore, the article explores the wide range of medical applications facilitated by reverse engineering, including prosthetics, implants, tissue engineering, and surgical planning. The potential of reverse engineering to enhance personalized medicine and patient-specific treatments is highlighted. A detailed proof of concept focusing on femur reconstruction is a significant component of the article. The proof of concept showcases the practical implementation of reverse engineering techniques to assist in designing and manufacturing precise custom-made implants and bone reconstruction. It emphasizes the integration of patient-specific anatomical data obtained through imaging technologies and the subsequent utilization of reverse engineering processes for anatomical reconstruction (solid modeling). Overall, this article provides an extensive overview of reverse engineering in medical applications, incorporating a literature review and a case study. The findings highlight reverse engineering’s potential to advance medical practices, improve patient outcomes, and foster personalized treatments. The review emphasizes the reverse engineering process, available technologies, and their specific relevance to the medical field, as well as their potential and effectiveness in advancing medical practices.

## Introduction

**Fig. 1:**
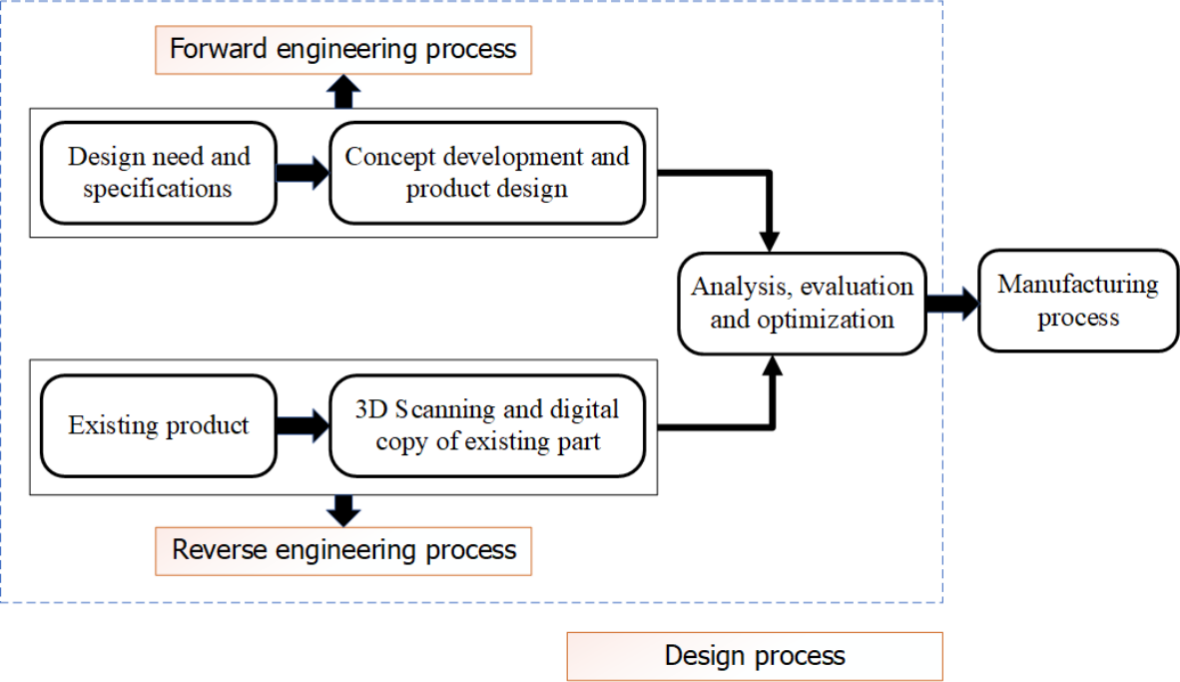
RE and traditional design process.

Reverse engineering (RE) is the technique of analyzing products to obtain their design information^1–3^. It is the opposite of the more common practice of forward engineering, which involves starting the production of a product with a systematic and logical design. RE has applications in a variety of sectors, including software, aerospace, automotive, defense systems, medical, consumer electronics, sports equipment, toys, and jewelry^3,4^. When reinventing a product, the RE process emphasizes analysis and assessment rather than creativity and originality, as seen in the design phase. Figure 1 depicts the main differences between RE and the traditional engineering design process in the context of the manufacturing process.

In the context of software, for instance, it could be extracting a source code from an executable file. When it comes to physical objects, RE is the digitization of physical products, i.e., obtaining a 3D surface geometry model to convert them to a computer-aided design (CAD) model.

Recent examples of reverse engineering in product development and replacement parts include product design and spare parts. In product design, it is used for creating new designs, especially when development starts from a physical prototype; making modifications during an iterative design process where modified products often lack CAD information; and verifying quality control by determining deviations in a design and comparing it with the original CAD model. For spare parts, reverse engineering is used for duplicating or producing original equipment manufacturer (OEM) parts; repairing or modifying parts without original design data; and generating models of existing parts that, for various reasons, lack CAD information.

To accurately create a physical object from a CAD model for medical purposes, advanced manufacturing techniques are necessary. With the development of new materials, reverse engineering is now better suited to digital models than to the traditional manual approach.

In the medical industry, the ability to generate 3D models from patient data using 3D scanners allows the creation and design of preoperatively suitable customized applications, body shape, implants, prosthetics, and anatomical models, surface texture, restoring shape and color safely. Broadly, in vivo and in vitro research are the two types of medical applications that utilize reverse engineering techniques. In vivo experiments are tests that use living organisms to mimic the simulated model, utilizing X-rays, MRI (Magnetic Resonance Imaging), and CT (Computed Tomography) scans. Such non-invasive procedures enable investigations to be conducted under natural living situations. Whereas, In vitro research refers to studies on samples that must be prepared prior to scanning. Typically, a 3D scanner is utilized to properly trace the complicated geometry of the sample, and to build the virtual model. Some of the applications include use in dentistry^5^and dental implantology^6^, prosthetic design^7^, organ fabrication^8^, forensic anthropology^9^, biomechanics^10^, neurosurgery^11^, surgical planning^12^, anatomical reconstruction of different organ parts^13,14^ and others. This method provides a revolutionary advance in surgical planning and simulation, based on patient-specific anatomy. Detailed applications of reverse engineering in the medical industry will be discussed in Sect. 4 of the article.

This article explores the use of reverse engineering in medical application based on a thorough literature review and a proof of concept to illustrate the practical applications. The focus is on how reverse engineering can enhance medical practices and improve patient outcomes. The review discusses the reverse engineering process, the technologies involved, and their relevance to healthcare, emphasizing the integration of advanced imaging techniques, such as computed tomography (CT) and magnetic resonance imaging (MRI), with reverse engineering to create accurate anatomical models. The article highlights various medical applications enabled by reverse engineering, including custom-fit dental implants, patient-specific medical devices, advancements in tissue engineering, and improved surgical planning. Another key feature of the article is a proof of concept on human femur model reconstruction, showcasing the practical implementation of reverse engineering. The study details how anatomical data was captured and used to design and manufacture a custom-made implant, exemplifying the potential of reverse engineering to produce precise medical solutions. Overall, the article underscores the significant impact of reverse engineering on advancing medical practices and improving patient care.

## Reverse engineering process and technologies

**Fig. 2:**
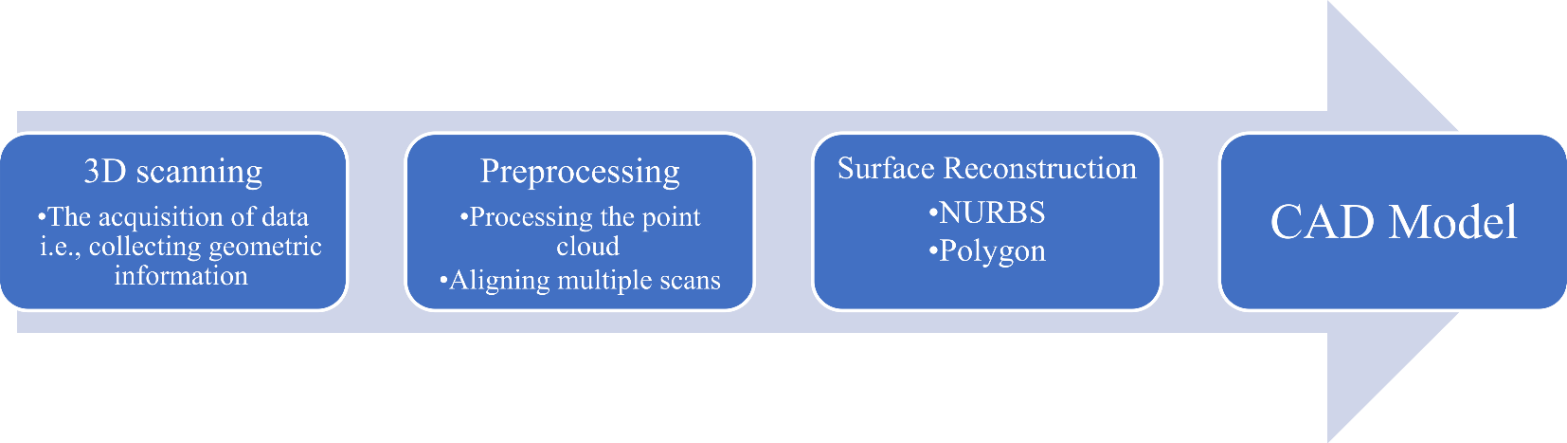
Reverse engineering process.

The process of digitization can be achieved through the utilization of 3D scanners, which are capable of capturing the physical attributes of an object, including its shape, size, and, in some cases, color. The initial stage of RE involves the acquisition of surface or geometric information pertaining to a particular object of interest. After the data has been acquired, it undergoes processing and is subsequently transformed into a three-dimensional model, thereby generating a digital replica. The possession of a digital copy facilitates additional modeling opportunities. The procedure is a conventional practice and is illustrated in Fig. 2. The data produced by contemporary scanning technology is amenable to software applications utilized for designing and analysis purposes. Raja and Fernandes^1^and Geng and Bidanda^4^ have compiled a comprehensive categorization of 3D scanning techniques.

**Fig. 3:**
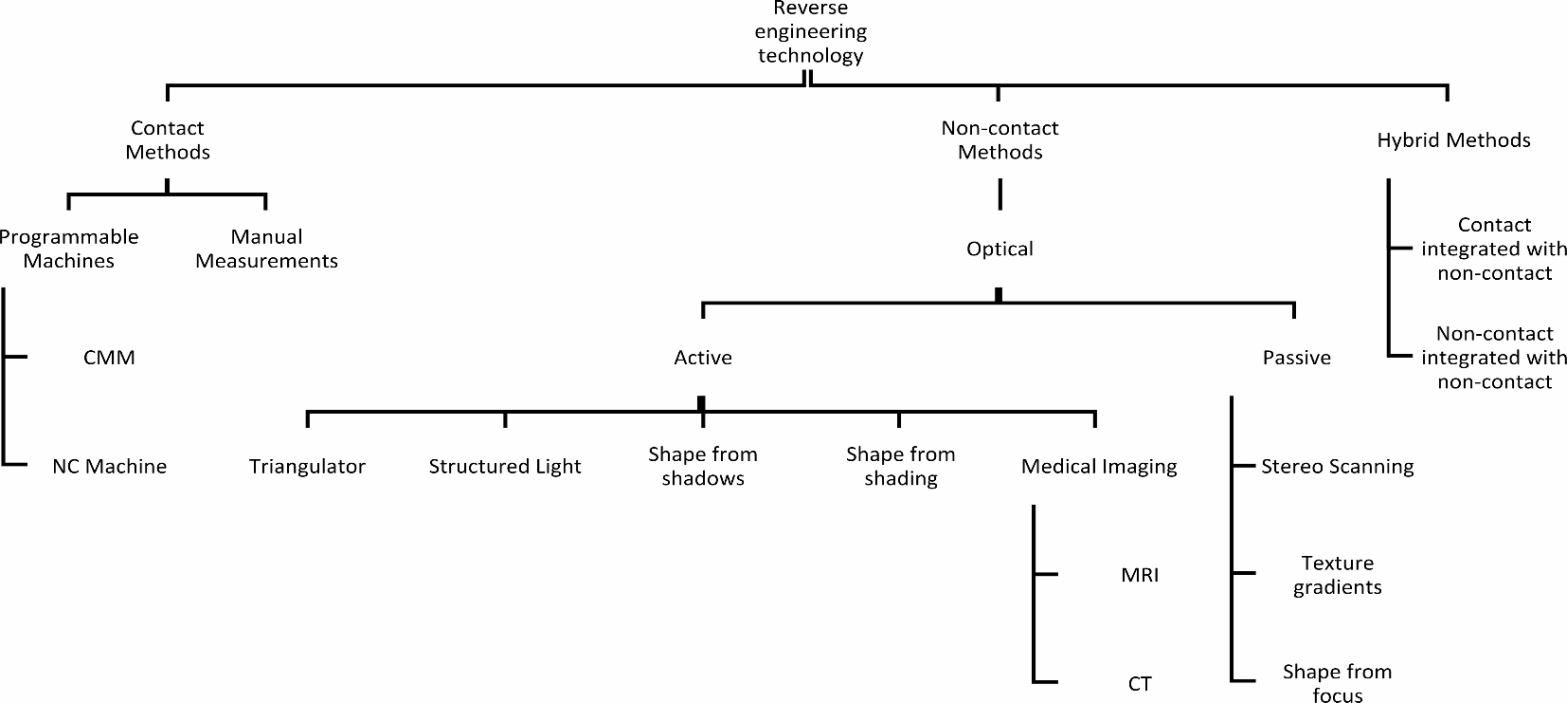
3D scanning classification^2^.

All scanning tools use a physical mechanism for the purpose of scanning, while the categorization of scanning technologies can be generally delineated by the presence or absence of physical contact with the object of interest^3^. Regarding this matter, scanners can be classified as contact, non-contact, or hybrid technology (Fig. 3).

Contact methods use a physical probe to travel along the surface of an object in order to collect information. This could involve manual measurement, coordinate measuring machines (CMM), or machines based on Numerical Control (NC). However, these methods are more time-consuming and are only applicable to objects with basic geometric shapes^4,15^. In addition, the sensors must maintain a particular contact pressure to accommodate very soft materials and tactile characteristics^16^. A contact probe’s sensing can be either point-to-point or analogous.

Non-contact methods involve no physical contact but, instead, utilize energy sources that project, such as light, laser, sound, magnetic fields, or X-rays^1–3^. Non-contact scanners are the method of choice for larger, more complex elements with multiple curvatures and free-form shapes. Capturing the return of this emitted energy (either transmitted or reflected) enables the determination of surface information.

Typically, an object must be prepared so that it can be scanned. Preparation can be either mounting or surface polish, for example, using white, temporary scanning sprays. It is essential to select an appropriate scanning technique or method. This ensures that all features of an object, e.g., holes, slots, pockets, and steps, are captured.

As shown in Fig. 2, the first stage is the 3D scanning phase. Using the appropriate technique, a 3D scanner is employed to scan a target object. Point cloud data is transferred to the pertinent software. In other words, discrete coordinates are used to approximate the object of interest during the digitization process. These are essential geometry references that will be used to define the geometry of the surface. 3D scanners employ various techniques to capture surface details from objects. Active and passive systems utilize distinct methodologies. The primary distinction between these systems is that active systems project energy and measure the transmitted or reflected energy to determine the geometry, whereas passive systems examine images of the geometry. These systems are based on the following physical principles^17^:

- Active Systems:
  - Triangulation.
  - Time-of-flight or laser pulse.
  - Interferometry.
- Passive Systems:
  - Shape-from-shading.
  - Shape-from-motion.
  - Passive stereo.

## RE and imaging techniques in healthcare

**Fig. 4:**
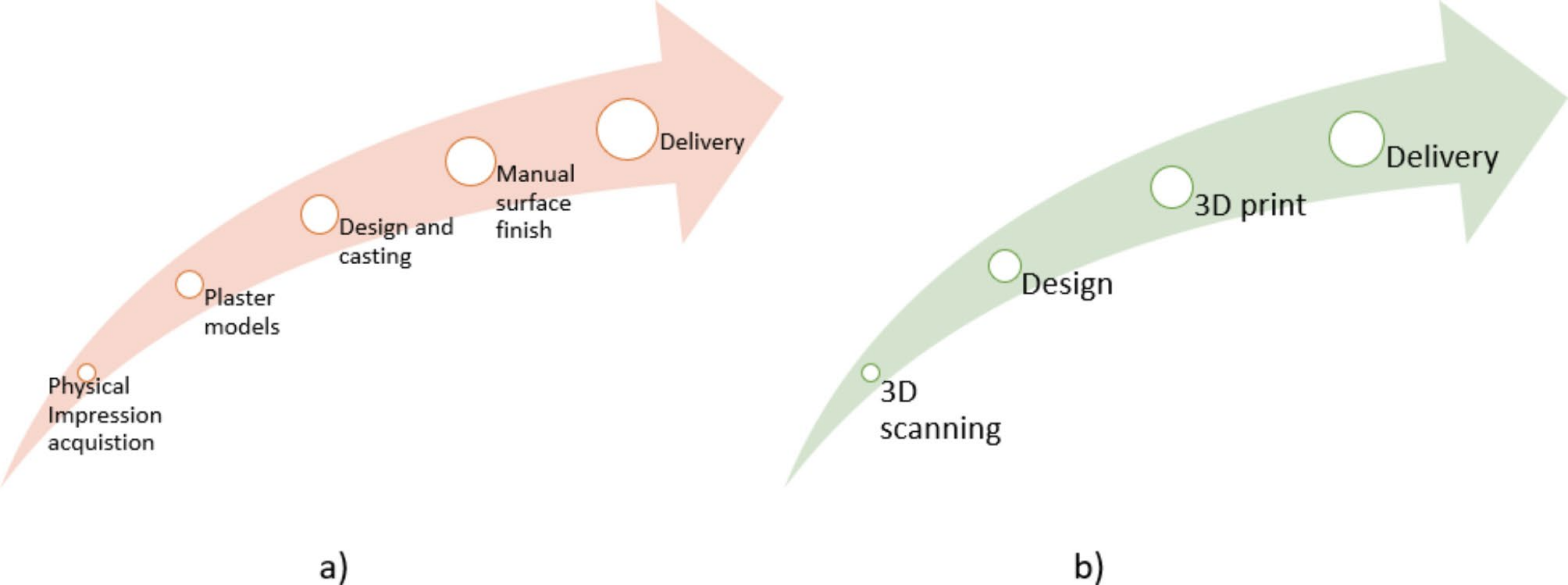
(**a**) Traditional workflow vs. (**b**) Digital workflow for dental applications.

The incorporation of digital solutions and the advancement of technology has revolutionized the techniques used for diagnosis and treatment planning. The utilization of three-dimensional scanning technology, particularly within the realm of dentistry, represents a significant breakthrough. The utilization of 3D scanners is advantageous in comparison to traditional methods for acquiring oral impressions such as plaster models that present various drawbacks^18^. Figure 4 displays the change 3D scanning technologies have created in the workflow within orthodontics.

3D scanning has changed cast model acquisition, record-keeping and storage, as well as other applications such as custom retainers and even surgery simulation. The utilization of extra- and intra-oral scanners has facilitated the achievement of these outcomes^19^. The feasibility of substituting (CT) scanning with non-contact 3D scanning technology, particularly laser scanning, has been proposed for accurate evaluations of implant surgery^20^. The objective of this methodology is to reduce the potential hazard of radiation exposure that could result from the utilization of a CT scanner. The study conducted by Taneva et al^18^. presents a thorough examination of the advantages and diverse methodologies utilized in 3D scanning.

The implementation of 3D scanning, in conjunction with additive manufacturing technology, has resulted in novel approaches for the visualization of anatomical structures within the realm of anatomy education. For instance, Di-Donato et al^21^. utilized 3D scanning tools for creating digital models of domestic animal tongues, which were then incorporated into practical classes as a means of facilitating instruction. An interlaboratory study was also conducted by Soodmand et al^22^. to compare the methods of scanning a femur in the context of medical image reconstruction. A comparative analysis was conducted between medical image reconstruction techniques utilizing CT scanning and optical scanning, with a 3D scanning approach serving as the reference model. The findings indicated that there is no significant difference between the CT scanned model and the 3D scanned model.

### Medical imaging techniques and technologies

Medical imaging, as seen in Fig. 3, is an active system of scanning. For diagnostic or therapeutic purposes, medical professionals can create images of both internal and external body regions. Medical imaging is a non-invasive technique for obtaining information about an injury or illness. Medical images provide information regarding the specific diagnosis and severity for quantitative and qualitative evaluations, as part of procedures performed by an appropriately trained medical radiology professional. The analysis of these images enables physicians to resolve clinical issues.

**Fig. 5:**
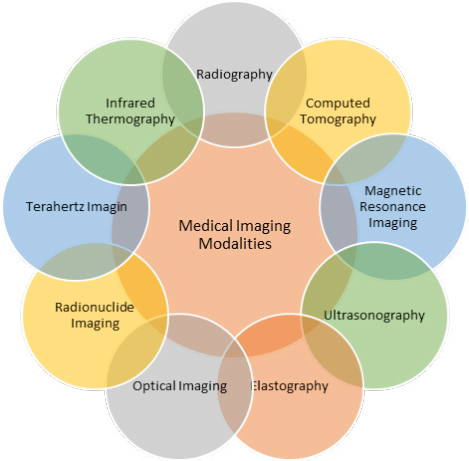
Medical imaging modalities.

Figure 5 demonstrates the most prominent imaging techniques. In the common medical format, the produced images are known as DICOM (Data Imaging and Communications in Medicine) format, and they contain information necessary for diagnosis.

Several works in the literature have provided details of the medical imaging modalities^23–25^. An overview of some of these modalities is highlighted below:

- ***Radiography***– This imaging modality involves the utilization of X-ray technology for the acquisition of two-dimensional planar images of anatomical structures. The majority of applications utilizing this technology pertain to the examination of fractures and alterations within the skeletal structure^23,24^. Radiography can be either fluoroscopy or projectional radiography^26^. Although low-cost and widely available, the quality of radiography images has been claimed to be poorer than the quality of images produced by CT and MRI^27^.
- ***Computed Tomography (CT)*** – In this technique, X-ray technology is utilized to acquire three-dimensional images of anatomical structures. Typically, the acquisition of a three-dimensional cross-sectional representation of the human body is facilitated by employing several X-ray sources and detectors.
- ***Magnetic Resonance Imaging (MRI)***– This technique utilizes the principle of nuclear magnetic resonance to facilitate both morphological imaging, such as soft tissue imaging, and functional imaging, such as blood flow imaging^23,24^. In contradistinction to CT, MRI radiation is non-ionizing^26^.
- ***Ultrasonography***– This is an ultrasound technique, which involves the application of acoustic waves exceeding 20 kHz; it is employed for diagnostic and occasionally therapeutic objectives. In the context of medical applications, the frequency typically falls within the range of 2.5–12 MHz. In contrast to other techniques, the utilization of ultrasound entails the absence of ionizing radiation, thereby precluding any potential biological harm. Additionally, ultrasound is a more cost-effective alternative to CT and MRI. Furthermore, this procedure enables imaging in real time, as stated in^26^.
- ***Elastography***– This is a technique that involves the diagnostic use of strain and elasticity tissue mapping. It is assumed that tissues that are harder or stiffer are unhealthy^26^.
- ***Optical Imaging***– The basic concept of this technique is the same as ultrasonic technology; however, it uses light rather than sound. Retinal imaging, cardiovascular imaging, gastrointestinal imaging, and dermatological imaging are only a few of the possible uses^23^.

## Medical applications of reverse engineering

The human body is a very complicated piece of engineering. As previously stated, reverse engineering can be regarded as a means of gaining knowledge in situations where there is a lack of data pertaining to an object. The human body can serve as a mechanism for the reinvention of its components, such as body parts and organs, through reverse engineering. This approach can be particularly useful in situations where there is an absence of design data, and it can facilitate a deeper understanding of the body.

Internal body components can be scanned using medical imaging, while external body parts can be scanned using 3D scanners. These tools facilitate the collection of information from a human subject, an attached device, or a model. When used in a medical setting, the steps depicted in Fig. 2can begin with a 3D scanning or medical imaging to learn more about a patient’s interior anatomy. Patient-specific preoperative 3D medical imaging data can be used in a wide variety of 3D bio-modeling and imaging applications, including the manufacture of implants, saw guides, and drill guides^28^.

Haleem et al^29^. conducted bibliometric analysis that displayed the increasing trend of researching 3D scanning applications in the medical field. This study’s main findings are:

- 3D scanning and printing are highly compatible and make a great medical support technology.
- 3D scanning offers insight into the external anatomy, while medical imaging explores more deeply the internal workings of the human body.
- All medical fields can benefit from the 3D scanning technologies.
- 3D scanning tools are reliable and helpful for carrying patient information electronically rather than physically (e.g., molds), provided that basic training is provided.
- For applications such as orthosis, prosthesis, and dentistry, 3D scanning offers a convenient replacement for the time-consuming process of making a plaster cast^30^.

The above research (Haleem et al^29^.) was driven by the demand for 3D scanning data for external body parts. RE is used in the medical field for a variety of medical applications, such as implants, patient shape, size, surface area, and body part information, as well as digital models for virtual reality and holographic applications. Implant manufacture is fast with a 3D scanner and post-process software. Additive Manufacturing (AM) is a method frequently used in RE. When integrated with AM technologies, RE has a wide range of potential uses^4,15,31^, including in dentistry, tissue engineering, and medical devices – all of which are further discussed.

### Dentistry

It appears that 3D scanners and printers are becoming increasingly commonplace in dental offices, although the intra-oral scanner is used to determine the amount of a damaged tooth in the most common routine check-ups. A variety of dental specialties, including prosthodontics, oral and maxillofacial surgery, oral implantology, and orthodontics, make use of RE and 3D scanners.

3D scanners provide surface information for dental preparations that are possible either in vivo or in vitro. The scanned data is used for implants or restorations. Recent applications of RE in dentistry include dental implants^32,33^, medical devices^34^, dental restorations^35^, and finite element method (FEM) on dental implants^36^. Dental impressions were traditionally taken using plaster models. However, the use of 3D scanners has made it possible to acquire digital impressions. The digital model’s dimensional precision and likeness match the plaster model^37^. RE dental devices include prostheses, implants, and surgical guides^33–35^.

**Fig. 6:**
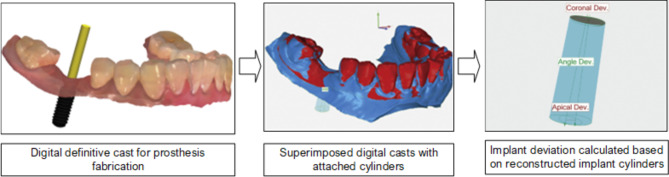
Procedures for evaluating the accuracy of guided implant surgery by using digital casts^40^.

RE has been implemented as a new method for comparing medical models produced by various 3D scanning techniques. This process was illustrated by Martorelli et al.‘s^38^research, in which Cone Beam Computed Tomography (CBCT) was used to scan the 3D model of simulated human dentition. Afterwards, the same 3D model was laser-scanned to make a comparison of the CBCT model with the laser-scanned model. Zhou et al^39^. conducted a study with a similar objective, analyzing and measuring dental casts and testing 3D scanning as a replacement for gypsum casts. Pereira et al^40^. and Liu et al^41^. conducted experiments examining the viability of digital casts, as well as the angle and distance between dental implants. These studies demonstrate the viability of using non-contact laser 3D scanning as an alternative to CT scanning for dimension verification, as well as deviation analyses based on the reconstructed implant cylinder. Figure 6displays the protocol for evaluating the accuracy of guided implant surgery by using digital casts^40^.

**Fig. 7:**
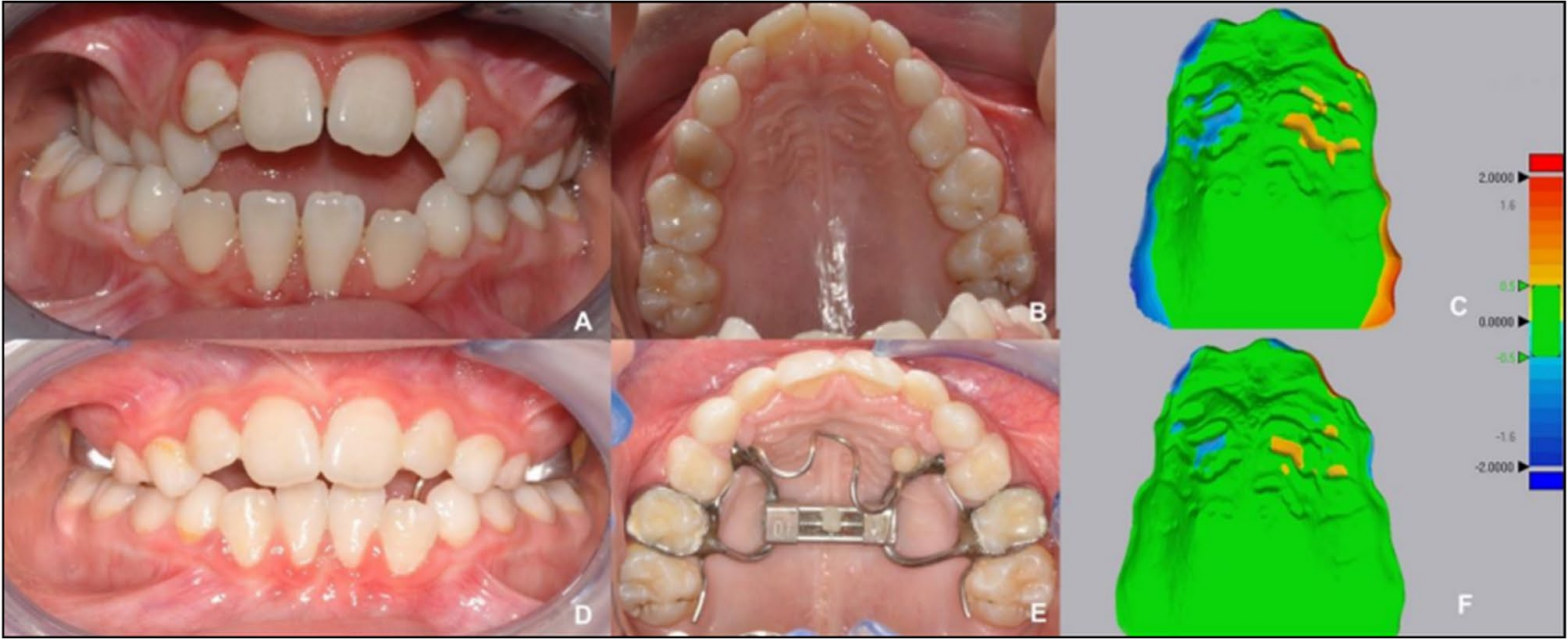
Computer-assisted analysis of maxillary asymmetry of a 7-year-old girl, using reverse engineering. (**A**) Pre-treatment frontal view, (**B**) Pre-treatment occlusal view, (**C**) Pre-treatment deviation analysis between original maxillary scan and mirrored scan, (**D**) Post-treatment frontal view, (**E**) Post-treatment occlusal view, (**F**) Post-treatment deviation analysis between original maxillary scan and mirrored scan.

Additionally, RE has been incorporated into dental analysis. For instance, Martorelli et al ^42^. utilized 3D models of the human mandible that were generated through RE. The objective was to recreate 3D models with the same or relatively similar mechanical properties as a human mandible. To ascertain the load-displacement relationship, the 3D model was then subjected to experimentation under suitable loading conditions as part of the validation procedure. In essence, RE was used in this study to ascertain the design information of the human mandible’s surface layout and strength. Additionally, finite element analysis (FEA) and fatigue analysis^43,44^were conducted for the same purpose. These studies demonstrate that non-contact laser 3D scanning can be used to obtain geometric data for analysis purposes. On the other hand, Giudice et al^45^. show that using the mirroring process and best-fit alignment algorithm, the differences between anatomical sides of the patient (left-to-right and right-to-left) can be obtained from the surface analysis and color map visualizations, as seen in Fig. 7.

The development of material science has led to the incorporation of composite materials into dental devices. RE has made it possible to analyze these composite materials. This is evident in the research conducted by Ilie^46^, which suggests that, through its analysis of clinically tested materials, RE is a viable method for future design. In this investigation, the materials analyzed were composites based on resin. In dentistry, RE tools enable kinematic analysis and virtual design in conjunction with CAD tools. This allows for the creation of a virtual articulator. A virtual articulator reduces the need for a mechanical physical articulator while allowing practitioners to simulate actual patient data and conduct analysis^47,48^.

**Fig. 8:**
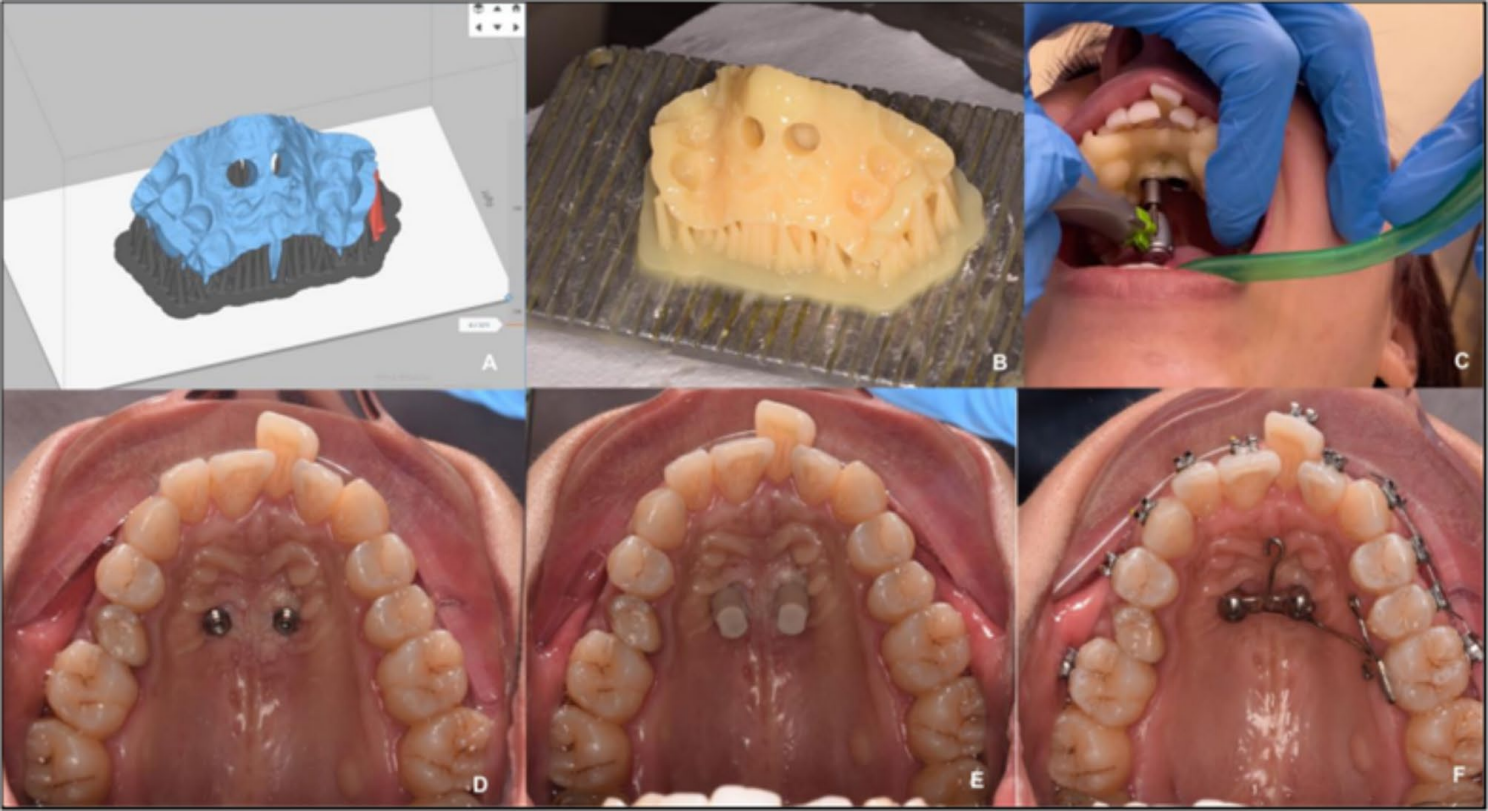
Clinical example of miniscrews’ placement with 3D printed guided systems. (**A**) Digital guide in the digital 3D printing plate, (**B**) 3D printed surgical guide, (**C**) Clinical procedure of miniscrew insertion, (**D**) Miniscrews placed in the palatal paramedian region, (**E**) Scan bodies for intra-oral scan, (**F**) Appliance fixed to the miniscrews.

The digital planning of skeletal anchoring systems is another way that RE is used in dentistry to overcome the constraints of traditional therapy. While a free-hand technique can safely implant mini-screws in this area, digital planning and the creation of surgical guides are recommended for the following reasons: The three main aspects of miniscrew management are as follows: (1) regulating miniscrew inclination and parallelism, to prevent undercuts and facilitate orthodontic appliance placement; (2) controlling miniscrew insertion depth, to prevent bone trauma during insertion; and (3) precisely planning the relationship between the miniscrew and the cortical palatal and nasal bone, which is essential for applying orthopedic forces^49^. Figure 8 describes a clinical example of miniscrew placement with 3D-printed guided systems developed from a reverse-engineered 3D model.

### Tissue engineering

Tissue engineering is a solution for regenerative medicine problems associated with tissue repair and organ replacement. 3D cellular structures (also known as scaffolds) provide an effective platform for the development of novel tissues, primarily by providing mechanical support during the development phase^50^. Scaffolds have an intricate structural composition. In this regard, RE plays a significant role as structural information for modeling, and it can be extracted using scanning techniques. In the research conducted by Pina et al^51^., multiple strategies that could be utilized to develop scaffold designs for tissue engineering and regenerative medicine for various organs and tissues are explained. The work describes the diverse applications of 3D scanning and RE for 3D printing hydrogel scaffolds. For instance, Li et al^52^. proposed 3D printed hydrogels as osteochondral (OC) defect fillers using alginate and hyaluronic acid as photopolymerized bioinks. The OC tissue was restored by reverse engineering, using high-resolution 3D scanning to obtain models of sample defects and the corresponding parts after regeneration. Wang et al^53^. distinguished between parametric design and the RE-based generation of vascular scaffold models. Due to the complexity of vascular scaffolds, it is said that parametric design without AI and machine learning is nearly impossible. However, parametric design in conjunction with AI and machine learning is said to be applicable for the bulk processing of models that are quite similar.

**Fig. 9:**
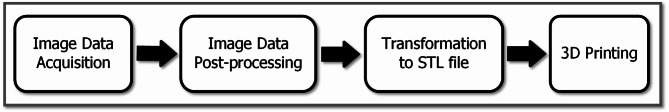
Workflow for creating 3D models from DICOM images.

The use of reverse-engineered models is applicable for patient-specific vascular scaffolds but is typically not employed for extensive quantity modeling where structural similarity exists. Figure 9depicts the workflow of 3D model reconstruction for these models, where MRI or CT images are used to acquire DICOM images. The general conclusion is that parametric design is more expedient, whereas RE models are more personalized and offer high anatomical compatibility^53^.

**Fig. 10:**
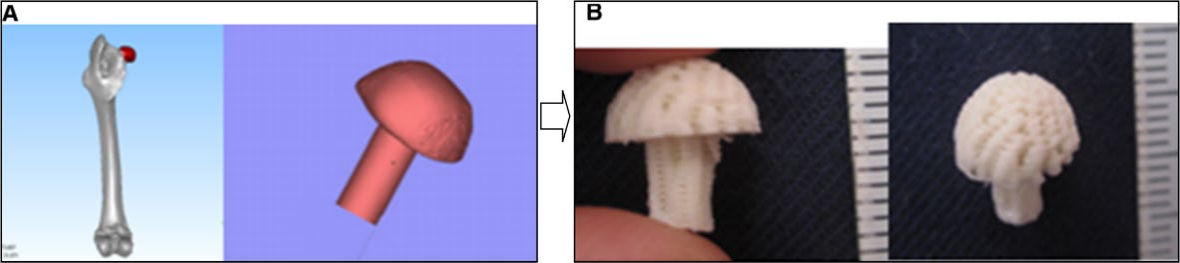
Designs of anatomical femoral and vertebral plate-fused scaffolds. (**A**) Design of rabbit femoral scaffold, (**B**) CT data-based rabbit femoral scaffold.

RE has also been applied to bone tissue engineering^54,55^. Again, RE is advantageous in the design and construction of scaffolds, in this case bone tissue scaffolds. After CT data of a bone is obtained, it is transferred to CAD software for design and analysis purposes. According to Yao et al^56^., animal (rabbit) spinal specimens were utilized to investigate design and construction procedures using RE, as seen in Fig. 10. Fucile et al^57^. demonstrated another application in this regard, where RE was used to create nanocomposite structures. In the work by Wang et al. ^58^, the RE process was implemented for bone tissue engineering, and the models were ultimately incorporated for computational fluid dynamics (CFD) to determine the flow field surrounding a cell.

### Medical devices

When the majority of a country’s debt is attributable to the import of medical devices, as was the case in the study by Asmaria et al^59^., the widespread adoption of RE can be an effective national strategy for debt relief. The research took place in Indonesia and involved the backwards engineering of an aneurysm clip (a highly sought-after medical device in that country). There have also been attempts to use RE in place of importing costly medical equipment in South Africa, another country with severe resource limitations^60^.

Recently, the use of RE during the COVID-19 pandemic has been quite prevalent. As the demand for personal protective equipment (PPE), ventilators, and other vital medical devices skyrocketed, supply chains began to break down. RE was proposed as a solution to these shortages, and numerous instances of hospitals developing equipment in-house to satisfy the high demand have been reported^61–64^.

These applications relate more to supply chain processes; nonetheless, they demonstrate the applicability of RE in the medical context. In the medical industry, RE can be said to have both direct and indirect applications. The aid RE provides in prosthetics and implants, surgical planning, customized medical devices, and anatomical modeling is among the direct applications of RE in the medical industry, whereas quality control, regulatory compliance, data integration, as well as research and development activities, can be categorized as indirect applications.

Surgical guides are important dental tools because they improve numerous aspects of the surgical procedure^65^. A CAD model produced with intra-oral 3D scanners is used to design and fabricate these devices. 3D scanning is used to collect intra-oral surface information. The accuracy of the surgical guide has been determined with the aid of RE and 3D scanning. One such instance is the work of Giordano et al^20^., in which RE was used to assess the precision of surgery guides in dental implants. In this way, RE is found to be a useful instrument for accuracy evaluation and quality control. Liang et al^66^. and Russo et al^67^. discussed additional applications of RE to ascertain the accuracy.

**Fig. 11:**
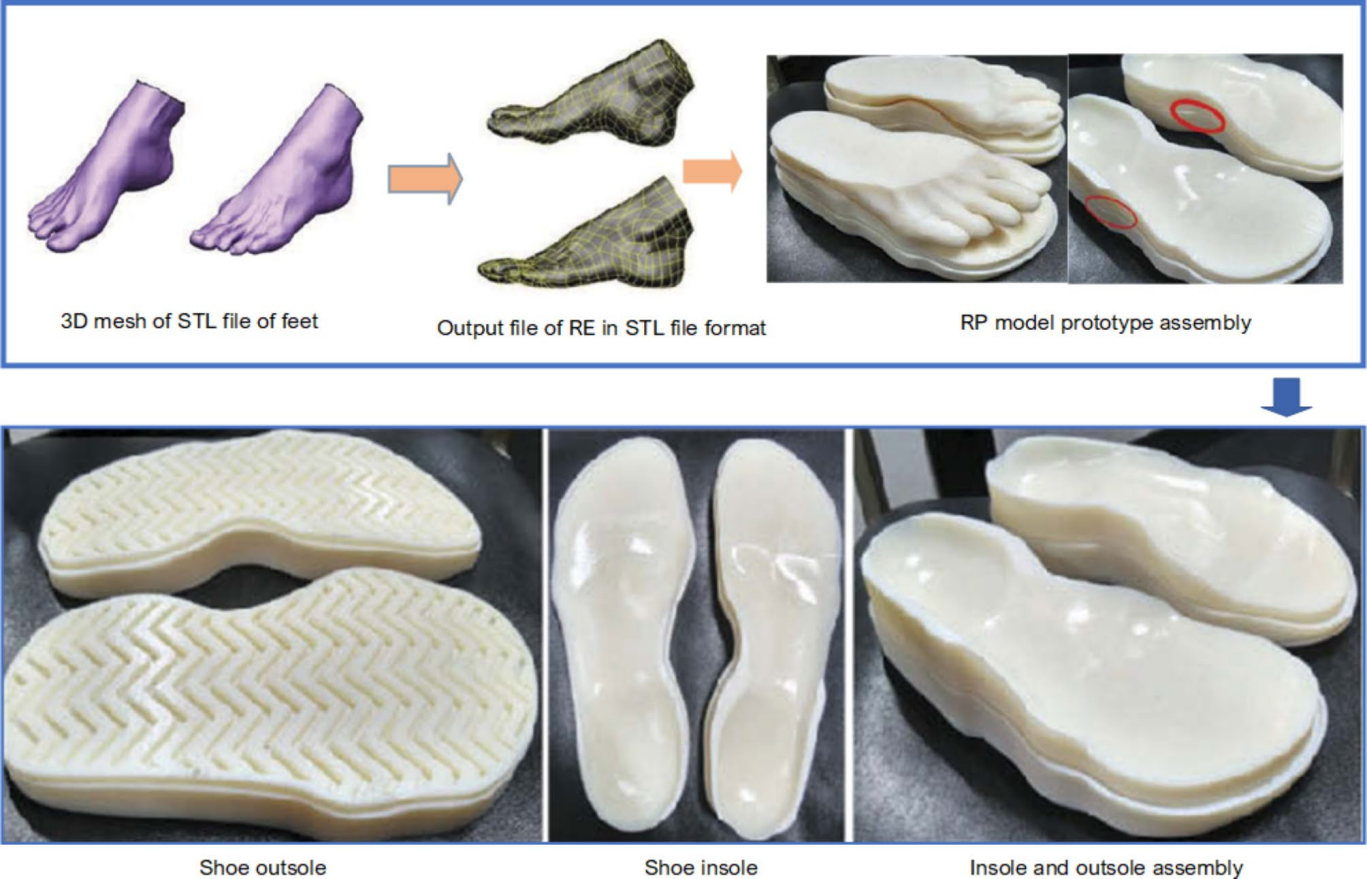
Design and production of orthotic insole shoes for diabetic patients^71^.

As previously stated, one of the opportunities provided by RE in the medical sector is the development of customized healthcare products. This enables customized implants and prosthetics for each patient. In their study, Noor et al^68^. demonstrated how a customized bone fracture implant developed from RE reduces the stress and movement between the fractured bone and the implant, in comparison to the implant constructed from a generic model. In addition to design and construction, RE has also been utilized in verification processes. In one instance described by Kloesel et al^69^., the RE procedure was used to verify the suitability of a medical device for a patient. Other uses for RE include hand orthotics^70^and soles^71^, as seen in Fig. 11.

**Fig. 12:**
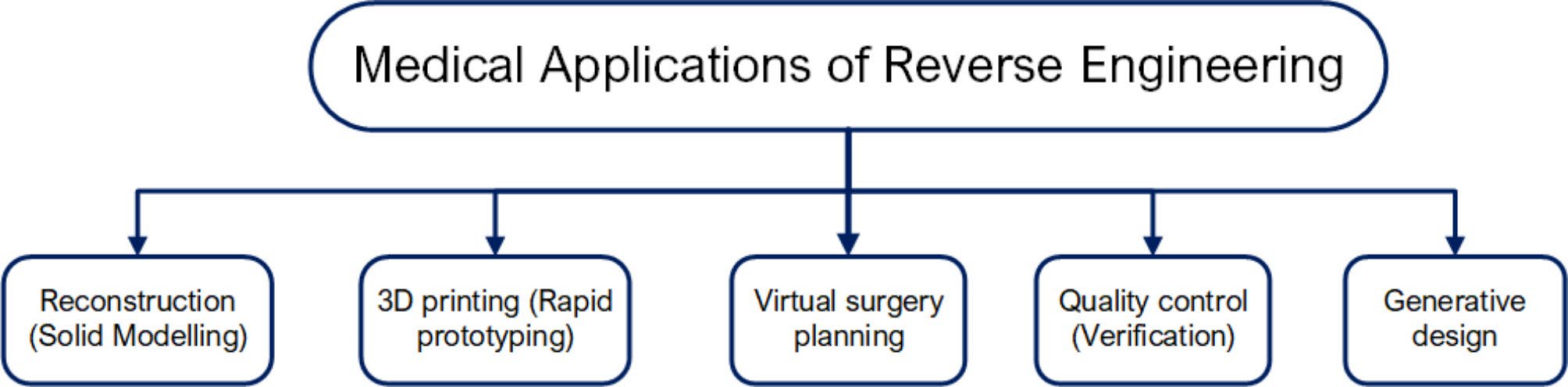
Summarized medical and biomedical application of RE.

Having gone through the various literature, some of the typical medical applications of RE are identified and summarized in Fig. 12.

The following section is a proof of concept on one of the above-mentioned applications of RE in the medical field, i.e., reconstruction (solid modeling). In this proof of concept, the protocol to reconstruct the human femur 3D model is provided. Additionally, the reconstructed model is compared to the reference model.

## A proof of concept: Femur reconstruction using RE

### Reconstruction processes and tools

As listed in Fig. 12, one of the medical applications of RE is solid modeling. In this section, the proof of concept of solid modeling for a human femur is conducted. The basis for the solid modeling is an STL file that is obtained from 3D scanning as a reference. Geometric modeling is frequently utilized today with the help of computational systems like CAD. The geometry of an object can be described in three ways: a wireframe model, where the shape is represented by lines and endpoints; a surface model, which includes lines, endpoints, and surface information with a mathematical description; and a solid model, which comprises lines, endpoints, surfaces, and volume information. A solid model can be used in a CAD system for various purposes, e.g., machining, FEA, etc. For medical applications, a solid model can be utilized for various purposes, such as for the design of implants and prostheses or for quality control, verification, and analysis. This is mainly because solid models provide properties such as mass and volume and can be used in mechanical assemblies. In the case of either designing or reconstructing medical applications, certain techniques are used to account for the missing information.

Several methods are utilized to design or reconstruct missing parts, to account for the lack of information in the medical field. This is not due to a lack of scanned data but, rather, to a lack of information regarding the presence of defective internal organs, bones, etc. One such study is mentioned in^72^, where current techniques are highlighted for the anatomical reconstruction of defective skulls. The methods described in this work include surface interpolation, deformed templates, mirroring, and sliced-based reconstruction. Surface fitting, contour skinning, volume polygonization, and implicit-function interpolation are generally the approaches that have been suggested for medical applications for designing and reconstructive purposes^73^.

**Fig. 13:**
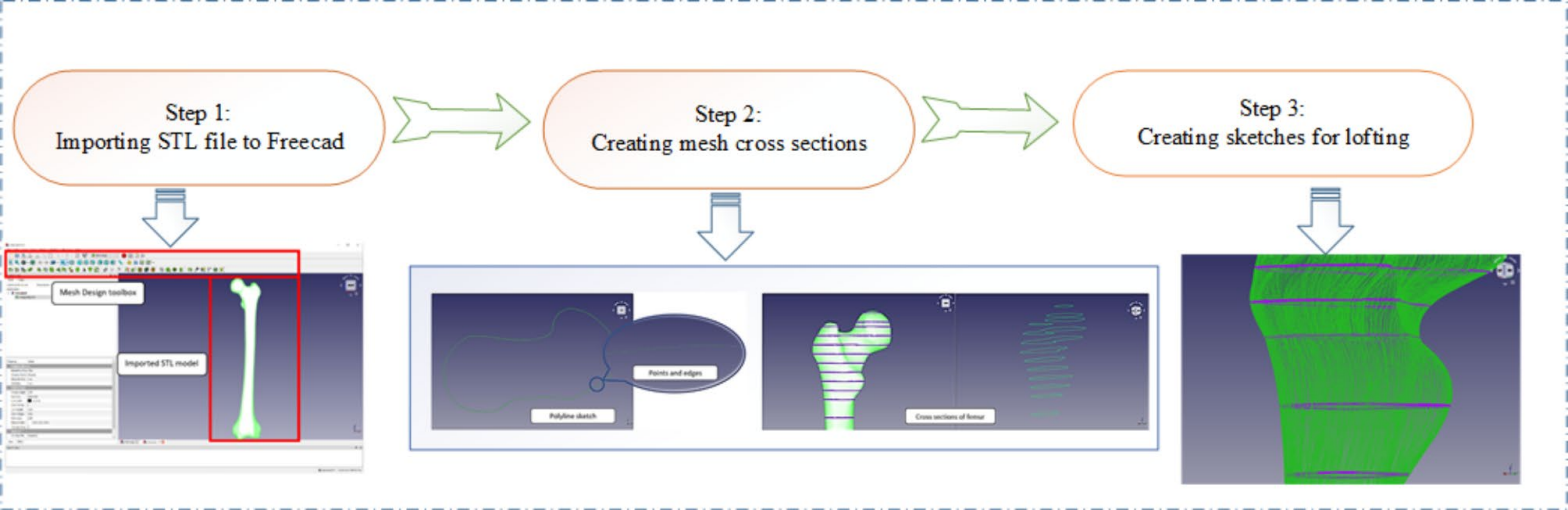
Major steps in the femur reconstruction process.

In this study, FreeCAD software was used. The approach involved directly lofting mesh cross-sections and using the FreeCAD workstation to turn each loft into a solid. Thereafter, a single solid model was created using Boolean functions following the procedure (steps) described in Fig. 13.

#### Step 1 - importing STL file to freecad

FreeCAD provides the importation of STL files for manipulation and has an entire workbench for mesh design and repair, as described in Fig. 7.

#### Step 2 - creating mesh cross sections

The mesh design workbench’s cross-section tool draws a polyline with model intersection points. Variables include section number, position, and connectivity tolerance.

#### Step 3 - creating sketches for lofting

Using sketches, loft between these cross-sections quickly. Loft with the polyline sketch. As mentioned, this sketch includes many edges, making lofting calculation more challenging. Lofting each section separately takes time. The algorithm that lofted each edge to a corresponding edge in the following sketch likely caused the improper interpolation between surfaces, as shown in the picture. If the curve points can be reduced without affecting geometric accuracy, lofting is better for simpler designs.

However, the sketch has numerous edges, which makes it more difficult to compute the lofting, and conducting individual lofts is time-consuming. One potential alternative is to convert the polyline drawing into a curved closed sketch with a single edge, which reduces the number of surfaces that must be interpolated during lofting. FreeCAD drafts of the workbench model include tools for constructing the curves; the available choices, including Bezier, cubic Bezier, and NURBS curves, will be considered to interpolate the current points in these curves.

**Fig. 14:**
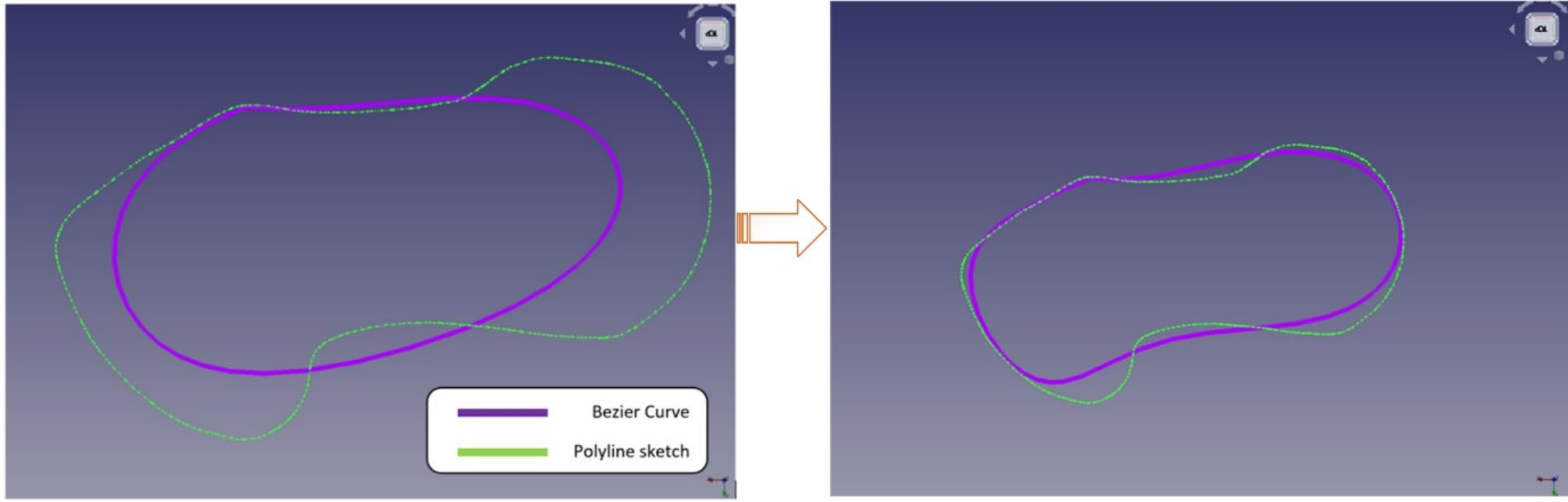
Sketch using Bezier curve (**a**) before adjustments (**b**) after adjustments.

The Bezier curve only traverses the polygon’s first and last points; otherwise, it follows the tangent of its other sides. Figure 14(a) shows the preliminary sketch created with the Bezier curve. This was created by combining points with a sketch. The images illustrate that the curve does not fit the profile of the polyline sketch. However, a sketch with a closed loop was created. Following the construction of the closed loop, the control points of the Bezier curve were changed to match the profile of the polyline sketch, as shown in Fig. 14(b). This figure (Fig. 14(b)) appears to be more accurate than Fig. 14(a), although it does not fit the polyline’s profile.

**Fig. 15:**
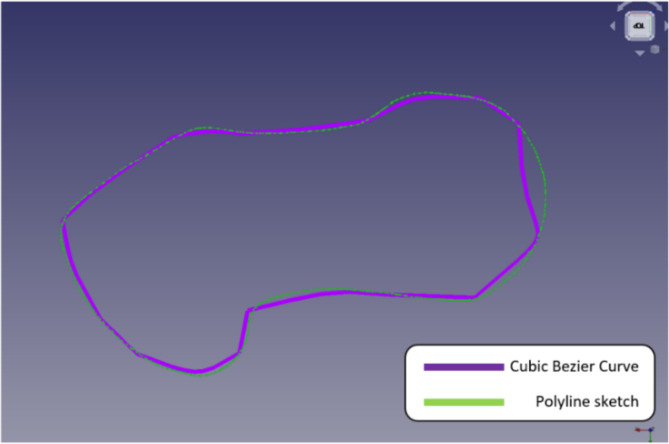
Sketch using cubic Bezier curve.

With this FreeCAD utility, three Bezier curves can be made from continuous splines of many degrees. Figure 15 shows that, while the cubic Bezier curve shows the design more accurately than the polyline sketch, the segments only give a rough outline. This was created by selecting points along with the sketch.

**Fig. 16:**
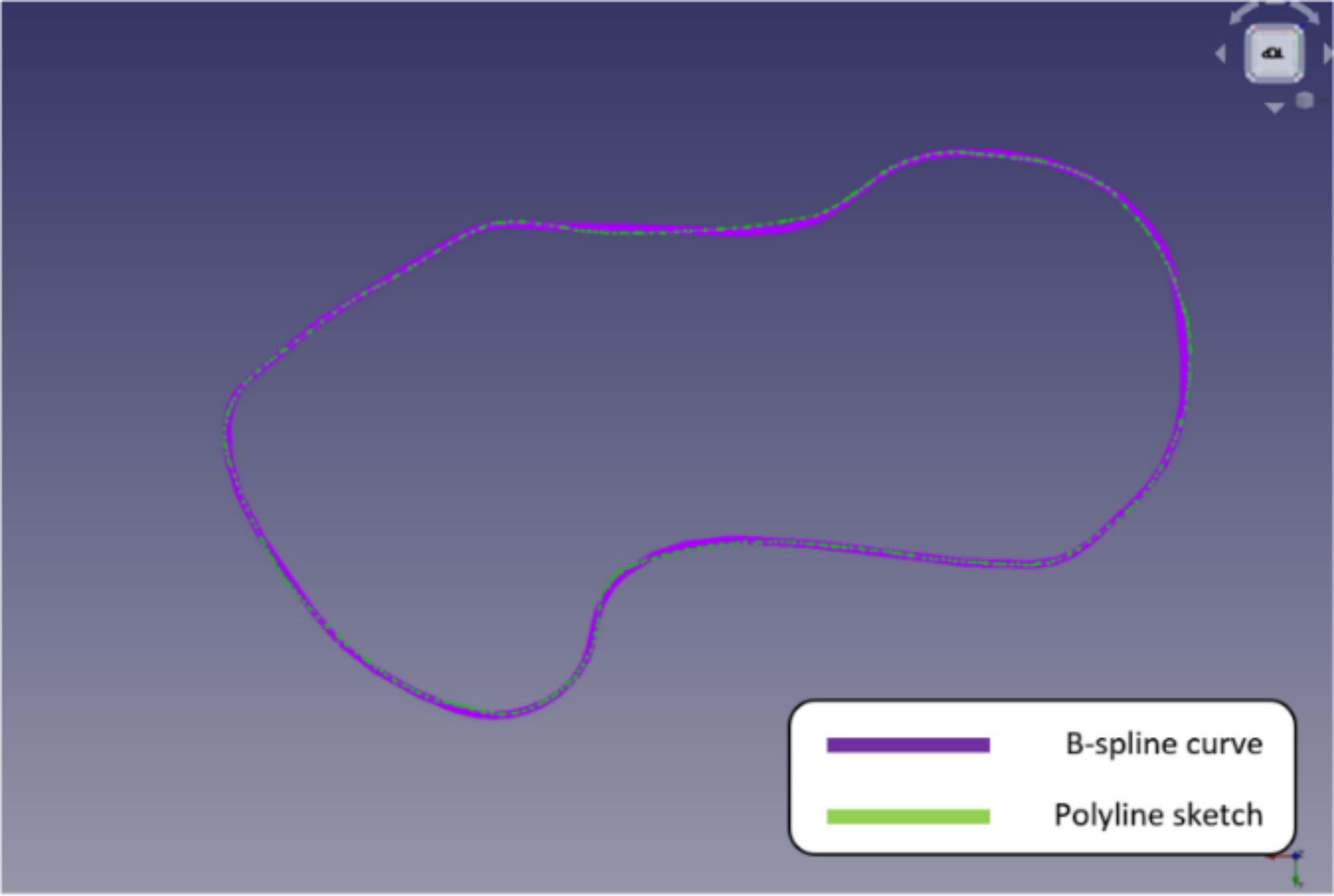
Sketch using B-spline curve.

B-spline curves can be adjusted locally, regardless of control point count. Figure 16 shows how well the cubic B-spline curve represents the sketch and how well the segments match the polyline sketch’s shape. To do this, selected points were added to the original sketch. Selecting points with the sketch produced this.

Using this technique, sketches are created manually because creating the contours automatically, using the points on the polyline sketch, will be an efficient alternative. Automatically generating the curves from the coordinates on the polyline sketch would be a suitable substitute. To achieve this, interpolation was performed between the polyline sketch’s points, using the points as references. The curves workstation of FreeCAD includes tools for interpolating between points. There are three possible methods: (1) connecting all edges with B-spline curves, (2) approximating points with a NURBS curve, and (3) interpolating between points using B-spline curves.

**Fig. 17:**
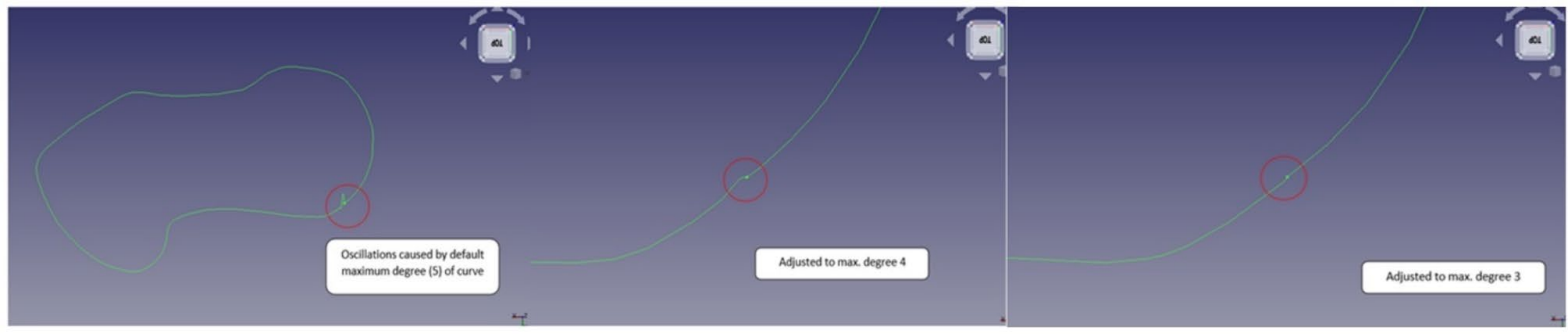
Sketch created using NURBS curve.

NURBS curves accurately represent conic curves, although B-splines only approximate them^74^. Yet, the FreeCAD program generates a roughly NURBS curve from the coordinates. FreeCAD produces an approximate NURBS curve from coordinates. To create a smooth, oscillation-free closed loop, this tool needs to be closed and the curve’s maximum degree changed. Figure 17 displays the initial curve and the subsequent revisions.

#### Interpolation using B-splines/join using B-splines

**Fig. 18:**
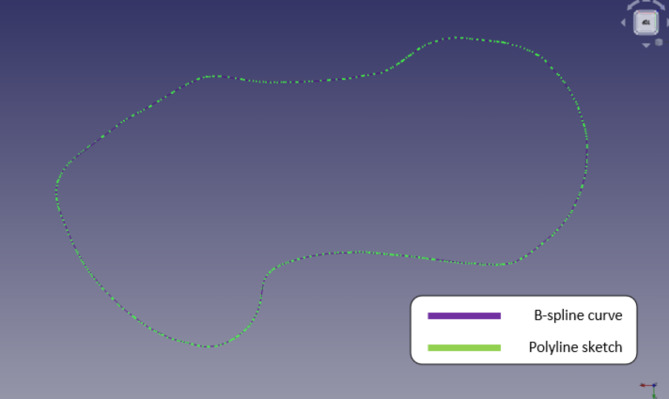
Sketch created by joining points using B-spline curves.

These functions differ in only two ways, and the interpolation does not close loops. A closed-loop sketch requires an additional step of setting the parameter “periodic” to “true”. The curve created by the join tool is not smooth. As shown in Fig. 18, the tools can build a closed-loop drawing from all polyline sketch locations.

**Fig. 19:**
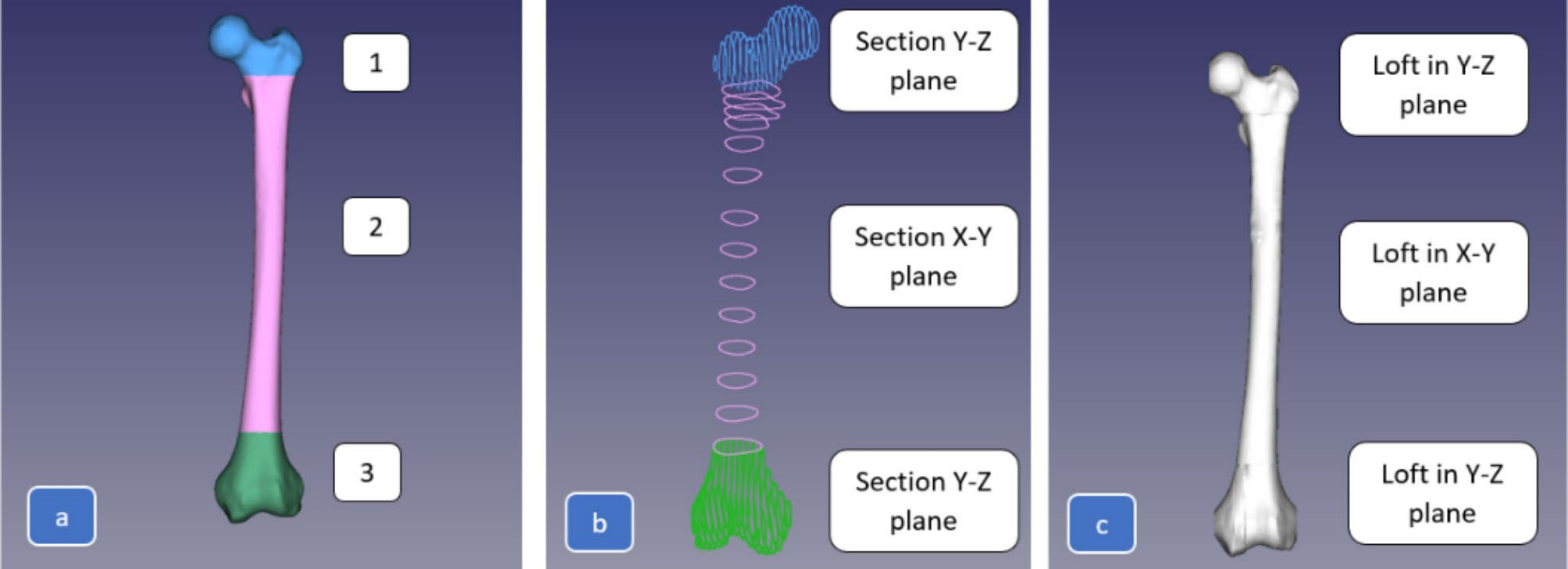
The full procedure for split mesh model of the femur.

A reliable femur model can be made after these three steps. Since the entire femur model does not need to be separated into portions in the same plane, separating the mesh while modeling may be more practicable. Three femur mesh model portions were cut (Fig. 19a). The trim mesh tool separates mesh models into polygons. After that, the first three steps were completed. Each mesh component was broken into smaller portions and turned into a closed-loop sketch utilizing interpolation, using points (Fig. 19b). Once enough interpolation curves were obtained, each segment was lofted to create a solid model (Fig. 19c).

B-spline surfaces are used in FreeCAD’s loft feature. Interpolation depends on sketch count. For more than ten drawings, the interpolation is of degree three; for less than ten, it is one less than the number of sketches. Thus, many solid lofts were created using these profiles and merged using FreeCAD’s union tool. Combining lofts with Boolean functions is more reliable. It is easy in FreeCAD, where the solid feature is first updated from “false” to “true” on the data tab, and then union in the part workbench can join lofts.

### Solid modeling results and discussion

**Fig. 20:**
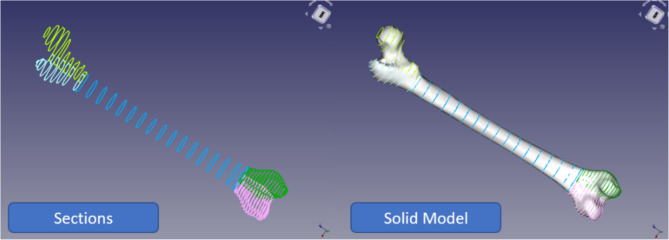
Final solid model of the femur.

Autodesk Inventor’s automated conversion capability produces a solid model that is compared to the approach outlined above in this section. The HandySCAN 700 3D scanner’s STL file was utilized to create a solid replica of the femur. Most of the geometry was missing from the original model. As illustrated in Fig. 20 (left), the femur model was further separated. This helped capture the femur’s features. For solid modeling in the CAD program, the HandySCAN model was selected. This was necessary to utilize as a benchmark for evaluating the final model built using the technique suggested in the study.

**Fig. 21:**
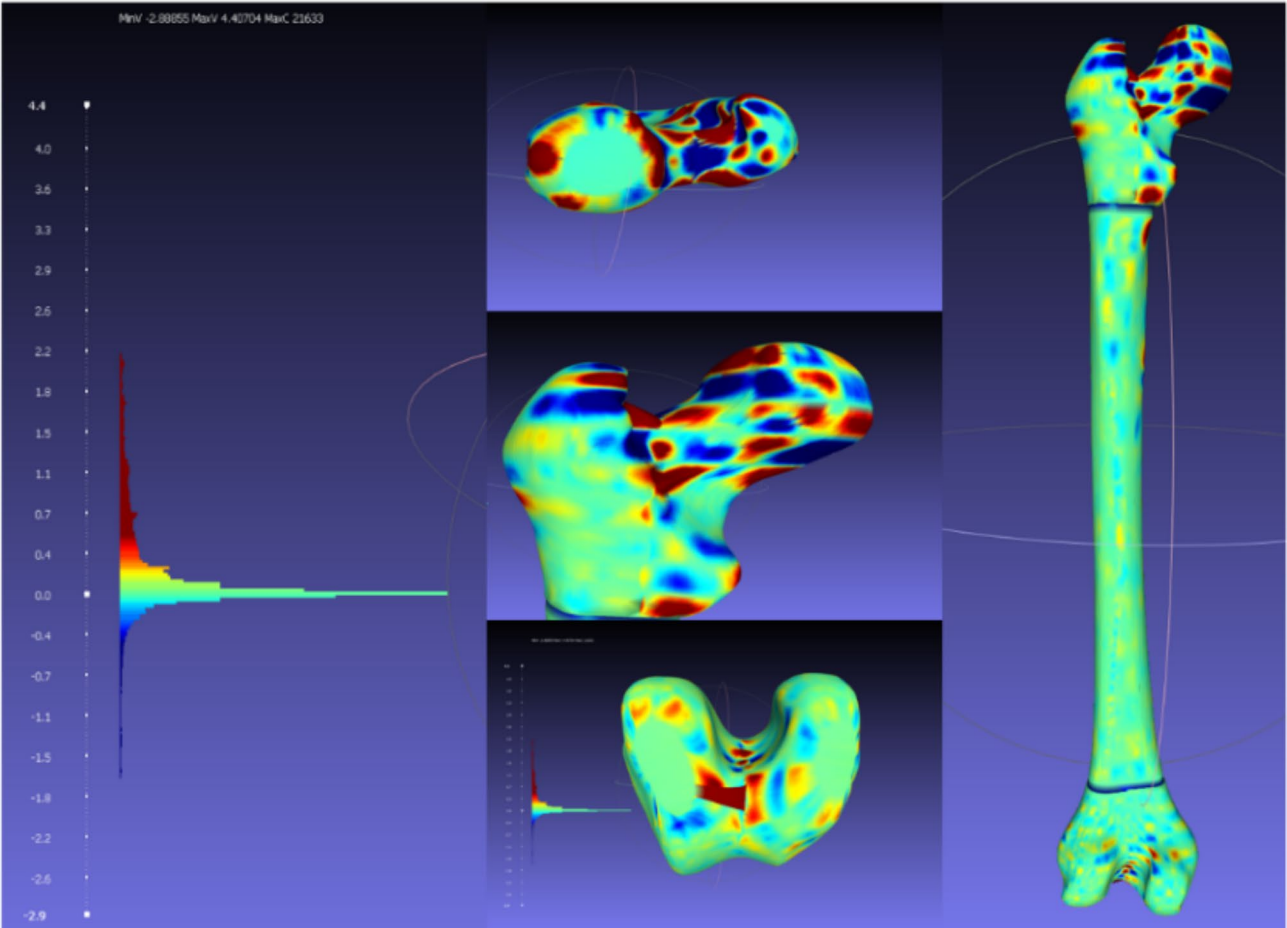
Final solid model vs. reference model.

The solid model was filtered, based on its distance from the reference mesh, as shown in Fig. 21. The referred-to STL file is not an exact representation of the desired model. Still, they are completely irrelevant.

**Fig. 22:**
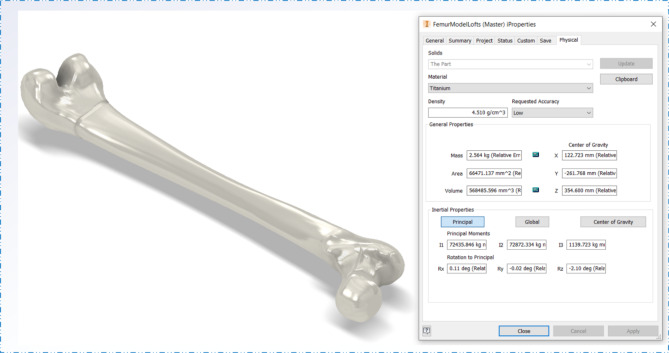
Solid model using FreeCAD.

**Fig. 23:**
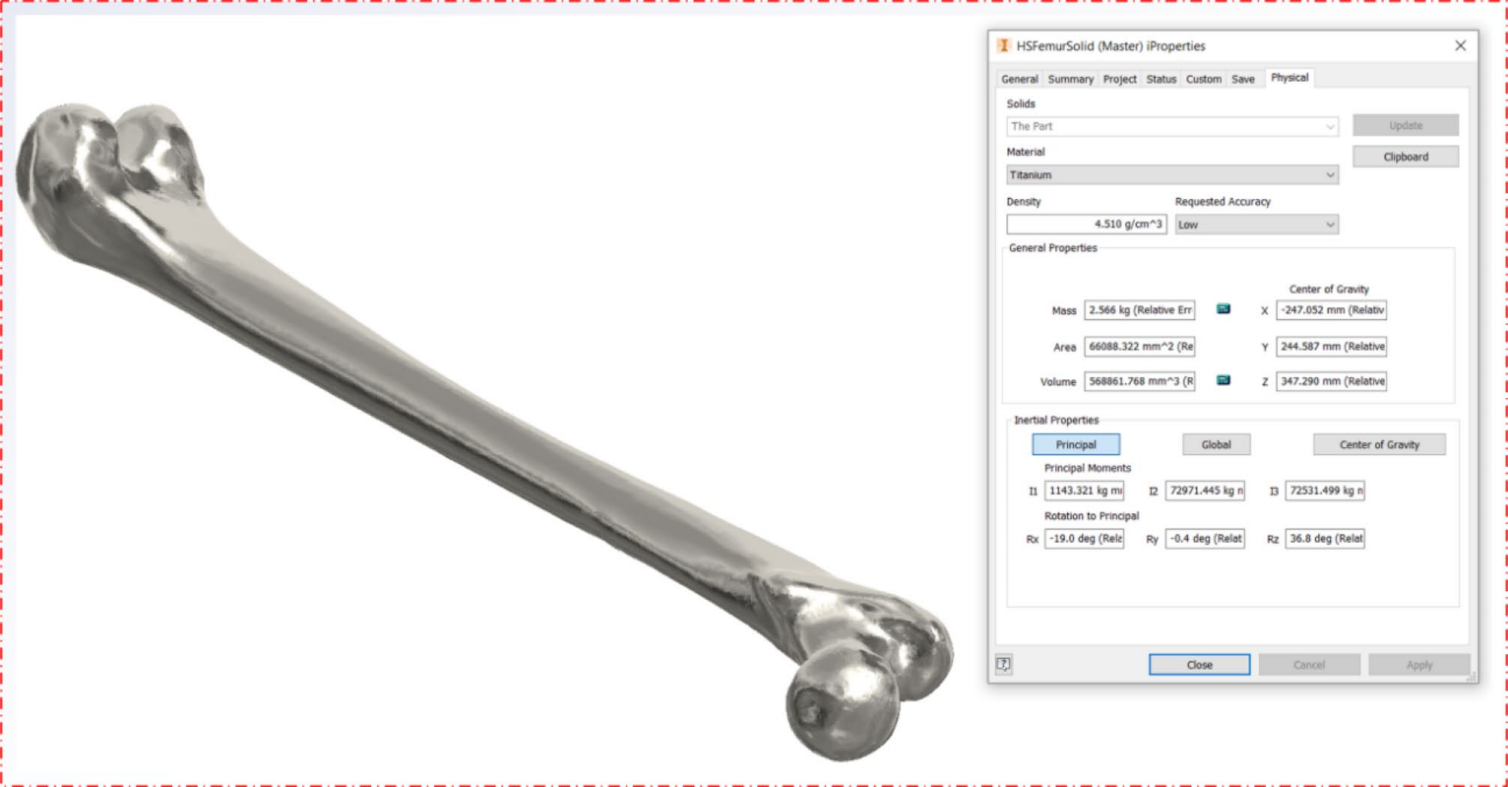
HandySCAN 700 model converted directly to solid in Autodesk Inventor.

For analysis in Autodesk Inventor, a STEP file was generated from the FreeCAD model and imported into the program. Titanium material properties were assigned throughout both the development (Fig. 22 - solid model using FreeCAD) and converted model (Fig. 23 - model from HandySCAN 3D scanner 700 converted directly to a solid model in Autodesk Inventor) so that their properties could be compared. The results are displayed in Table 1 and in Figs. 22 and 23.

**Table 1:** FreeCAD vs. Autodesk model.

	FreeCAD Model	Autodesk Model
Weight (kg)	2.564	2.566
Volume (mm^3^)	568,485.596	568,861.768
Area (mm^2^)	66,471.137	66,088.332

Table 1 shows the results of the comparative analysis, demonstrating a close similarity between the FreeCAD and Autodesk models in terms of weight, volume, and surface area. The weight of the FreeCAD model was 2.564 kg, while the weight of the Autodesk model was slightly higher at 2.566 kg. This marginal difference of 0.002 kg (about 0.08%) suggests that the two models are very similar in terms of mass.

Similarly, the FreeCAD model had a volume of 568,485.596 mm^3^, whereas the Autodesk model had a slightly bigger volume at 568,861.768 mm^3^. This variation of around 376.172 mm³ (approximately 0.07%) highlights the consistency between the models in representing the femur’s spatial occupancy.

The analysis of surface area reinforces the similarity between the models even further. The FreeCAD model had a surface area of 66,471,137 mm^2^, while the Autodesk model had a surface area of 66,088,332 mm^2^. This difference of approximately 382.805 mm^2^ (approximately 0.58%) highlights the similarity of their geometric properties in general.

The remarkably small percentage differences across all metrics demonstrate the robustness of both the FreeCAD and Autodesk methods for generating accurate solid femur models. These results validate the efficacy of the proposed technique in generating a reliable solid model for future medical applications and highlight the potential for using reverse engineering methods in medical research and practice.

## Discussion and future outlook

Upon reviewing the existing literature concerning the utilization of reverse engineering within the medical domain, many conclusions may be drawn. The medical industry utilizes the process of RE for both direct and indirect applications. This pertains to the utilization of applications that are employed directly on patients for surgical procedures, for clinical studies, and for research. The industry also benefits from indirect applications that aid in the facilitation of supply chain processes. Among these technologies, 3D scanning stands out as a particularly effective tool for supporting medical applications. The utilization of 3D scanning presents a feasible option for acquiring impressions in various applications, such as orthotics, prosthetics, and dentistry. Additionally, the process of RE can be applied to conduct analyses, such as FEA and CFD. Also, it enables the analysis of motion and the creation of virtual designs.

According to the femur reconstruction proof of concept study, the results demonstrate the effectiveness of the proposed technique for generating a solid femur model that closely matches the HandySCAN reference model. The minute variances in mass, volume, and surface area, all less than 0.1%, demonstrate the dependability and precision of the FreeCAD and Autodesk Inventor conversion processes. This not only validates the method’s viability but also highlights the potential for consistent and dependable outcomes in comparable medical and engineering applications.

Reverse engineering in medicine is expected to increase significantly in the future, due to growing interdisciplinary collaboration and technological advancements. The potential for innovation and improved healthcare is enormous, when biologists, engineers, and medical experts collaborate. Reverse engineering presents a singular chance to comprehend and reproduce intricate biological structures, resulting in ground-breaking discoveries in disciplines like regenerative medicine. Through reverse engineering approaches, significant advancements could be made in tissue engineering and organ replacement, for example, which depend on the accurate replication of biological systems by aiding in the development of precise structural frameworks for tissue scaffolds. These advancements have the potential to alleviate the severe lack of transplantable organs and enhance patient outcomes, by precisely imitating the complex architecture of tissues and organs.

Looking ahead, the continued progress of reverse engineering in medicine is anticipated to result in more personalized and effective healthcare solutions. As science and technology progress, the potential to tackle complicated medical problems using reverse engineering will grow, opening up new avenues for treatment and prevention. By addressing ethical concerns and encouraging cross-disciplinary collaboration, reverse engineering has the potential to shape the future of healthcare, making it more inventive, efficient, and suited to particular patient requirements. This breakthrough will be critical in addressing some of the most difficult issues confronting modern medicine.

## Data Availability

The datasets used and/or analysed during the current study available from the corresponding author on reasonable request.
